# Impact of Resuscitated Cardiac Arrest in the Brain-dead Donors on the Outcome of Liver Transplantation: A Retrospective and Propensity Score Matching Analysis

**DOI:** 10.1097/AS9.0000000000000522

**Published:** 2024-11-25

**Authors:** Shengmin Mei, Jie Xiang, Li Wang, Yuan Xu, Zhiwei Li

**Affiliations:** From the * Department of Hepatobiliary and Pancreatic Surgery, the First Affiliated Hospital, Zhejiang University School of Medicine, Hangzhou, China; † Department of Surgery, Zhejiang Hospital, Hangzhou, Zhejiang, China.

**Keywords:** cardiac arrest, donation after brain death, ischemic precondition, liver transplant

## Abstract

**Objective:**

To evaluate the impact of cardiac arrest time (CAT) in brain-dead donors on graft and recipient outcomes following liver transplantation.

**Background:**

The outcome of livers from brain-dead donors with a history of cardiac arrest (CA) remains controversial, and the duration of the CAT has never been evaluated.

**Methods:**

A retrospective review of data from the Scientific Registry of Transplant Recipients between 2003 and 2022 was conducted. Propensity score matching was performed to minimize confounding effects.

**Results:**

A total of 115,202 recipients were included, 7364 (6.4%) and 107,838 (93.6%) of whom were of the CA and non-CA group, respectively. After 1:1 propensity score matching, each group consisted of 7157 cases. The CA group demonstrated shorter hospital stay (15.5 ± 20.0 days vs. 16.2 ± 21.3 days, *P* = 0.041), with comparable incidence of early graft failure (EGF, 5.8% vs. 6.2%, *P* = 0.161). The CA group demonstrated slightly higher graft survival rates (1 year, 90% vs. 88%; 5 years, 76% vs. 74%; and 10 years, 61% vs. 58%, *P* < 0.001). CAT positively correlated with EGF [odds ratio (OR) = 1.03, 95% confidence interval (CI) = 1.02–1.04, *P* < 0.001], with a sensitivity and specificity of 73% and 86% at a cutoff of 30 minutes. The CAT <30 minutes group demonstrated significantly lower incidence of EGF (5.0%), compared with 7.8% of the CAT >30 minutes group and 6.2% of the non-CA group (*P* < 0.001).

**Conclusions:**

The use of brain-dead donors with a history of CA did not increase the risk of liver graft failure in our study. A downtime of <30 minutes may confer protective effects on transplanted grafts.

## INTRODUCTION

Liver transplantation (LT) remains the most effective treatment for end-stage liver disease and carries the potential to significantly improve patient survival and quality of life.^1^ However, the global shortage of donor livers has been a major obstacle to the development of this field.^2^ Various measures have been implemented to expand the donor liver pool and alleviate the imbalance between organ supply and demand. This has involved the expansion of the donor criteria and the utilization of donors after circulatory death.^3–5^ Such marginal donors are considered to possess characteristics that can negatively impact graft function and recipient prognosis, such as advanced age, increased degree of steatosis, and prolonged cold ischemia time. Balancing the risks of marginal donor livers against the clinical profile of recipients, and the development of rational donor assessment and selection strategies are thus highly prioritized in the field of LT.^6,7^

Donation after brain death (DBD) represents the primary organ source in most countries, given the ease in diagnosing brain death and the ability to control the timing of organ procurement. DBD livers accounted for 92.1% of all LTs in the United States in 2019.^8^ However, following the establishment of brain death, cardiac arrest (CA) necessitating cardiopulmonary resuscitation for restoration of spontaneous circulation can still occur. While reversible, such events can result in prolonged warm ischemia time, hemodynamic instability, and metabolic disturbances, increase the susceptibility of grafts to ischemic-hypoxic injuries, and subsequently affect transplant outcomes.^9–15^

Current understanding of the impact of donor CA on LT outcome remains limited, and many transplant centers remain cautious about the use of cardiac-arrested donors. There is increasing recognition that brief ischemic episodes induced by CA may exert protective effects on liver grafts, a phenomenon known as ischemic preconditioning.^9,16,17^ Current available studies involving DBDs with resuscitated CAs are scarce, mostly single-centered and retrospective in design, and involve small sample sizes.^9,16,18,19^ As such, the safety and durability of livers from donors of such profile, as well as the extent of ischemic preconditioning that is beneficial, remain largely unclear.

This study thereby aimed to evaluate the clinical utility of liver grafts from DBDs with a history of resuscitated CA using large-sample data and propensity score matching (PSM) and to identify factors associated with graft and recipient prognosis, with a particular focus on CA time.

## METHODS

### Data Source

This study used data from the Scientific Registry of Transplant Recipients (SRTR). The SRTR data system includes data on all donor, wait-listed candidates, and transplant recipients in the United States, submitted by the members of the Organ Procurement and Transplantation Network (OPTN). The Health Resources and Services Administration (HRSA), US Department of Health and Human Services provides oversight to the activities of the OPTN and SRTR contractors. The entire study was reviewed and approved by the Ethics Committee of the First Affiliated Hospital, School of Medicine, Zhejiang University, China.

### Study Population

**FIGURE 1.**
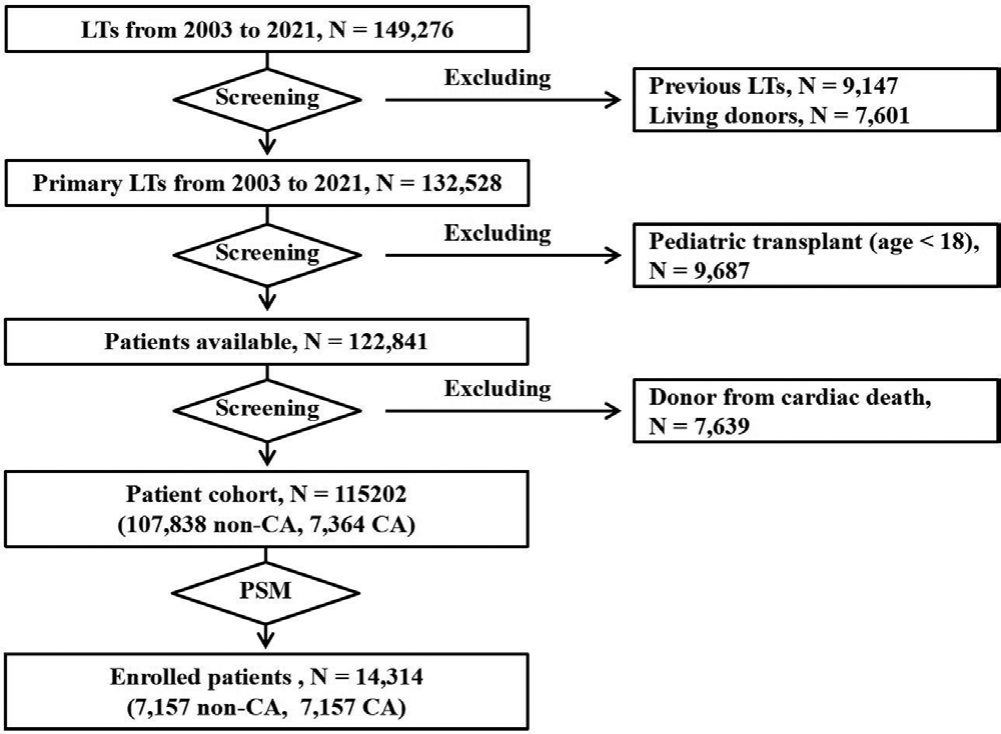
The subject selection process.

The study population included all adults who underwent DBD LT from the United States between January 1, 2003 and December 31, 2022. The subject selection process is shown in Figure 1. This study focused on preprocurement CA, which is defined as requiring cardiopulmonary resuscitation from the time of first contact between the patient and the medical team/inpatient department, and the organ procurement surgery in DBD. Cardiac arrest time (CAT) was defined as the total duration of cardiopulmonary resuscitation.

### Covariates

The demographic, clinicopathological, and transplant-specific variables of both donors and recipients were collected. Recipient-related factors collected included age, race, sex, body mass index (BMI), etiology of liver disease, model for end-stage liver disease score, hospitalization status at transplantation (hospitalized, in intensive care unit, or not hospitalized), medical history (hypertension, diabetes, portal vein thrombosis, or spontaneous bacterial peritonitis), history of abdominal surgery, and duration of hospital stay. Liver disease was divided into the following for stratified analysis: hepatitis B virus-related cirrhosis, hepatitis C virus-related cirrhosis, hepatocellular carcinoma, alcoholic liver disease, nonalcoholic steatohepatitis, and others.^20^ Donor-related factors collected included age, race, sex, BMI, cause of death, medical history (diabetes and hypertension), ischemia time (cold and warm), laboratory results (aspartate aminotransferase (AST), alanine aminotransferase (ALT), total bilirubin, international normalized ratio, serum sodium and creatinine levels), donor risk index (DRI), and type of LT (whole or partial graft). The cause of death was categorized as asphyxiation, cerebrovascular accident/stroke, head trauma, or others. DRI was calculated according to the formula established by Feng et al.^21^ The donors were divided based on history of CA (the CA and non-CA groups).

### Outcome Measures

The primary outcomes included early graft failure (EGF), graft survival, and patient survival. Preexisting conditions and the time of outcome occurrence were assessed. Recipient follow-up time was defined as the time from transplant surgery to death or the last known follow-up date, whichever occurred first.

The occurrence of recipient death and graft failure was determined based on data provided by the transplant centers, the US Social Security Administration, and the OPTN. EGF was defined as graft failure occurring within 90 days after transplant surgery. Graft survival time was defined as the time from transplant surgery to graft failure or patient death. The date of graft failure was defined in the SRTR as the date of patient relisting for transplantation or death. All recipients with functioning grafts at the last follow-up were censored. The cause of graft failure was classified as primary non-function (PNF), vascular thrombosis, hepatitis, rejection, disease recurrence, death with functioning graft, and unknown. Causes of death included graft failure, cardiovascular problems, organ failure, infection, malignancy, others (such as bleeding and trauma), and unknown.

### Statistical Analysis

Continuous variables were presented as mean ± standard deviation, while categorical variables were presented as number (percentage). Intergroup comparisons were performed using one-way analysis of variance and *χ*^2^ test, respectively. Graft and patient survival analyses were performed using the Kaplan–Meier method, with intergroup comparisons made using the log-rank test. The impact of CA on graft and recipient outcomes was assessed using the Cox proportional hazards model. Considering the large-sample size, we assessed both the statistical significance and clinical relevance of the results. Effect sizes, including odds ratios (ORs) or hazard ratios (HRs) with 95% confidence interval (Cis), were used to evaluate the magnitude of between-group differences. All statistical analyses were performed using the SPSS 25.0 software, with *P* < 0.05 considered statistical significance.

### Propensity Score Matching Analysis

PSM was performed to minimize the impact of potential confounding factors on transplant outcomes. The PSM model was established by the use of statistically significant covariates from the multivariate logistic regression analysis. A 1:1 nearest neighbor matching algorithm was used with a matching tolerance of 0.02.

The final model with EGF as the dependent variable is shown in Supplemental Table 1, http://links.lww.com/AOSO/A434. The matched donor-related covariates included age, sex, BMI, hypertension, diabetes, cold ischemia time, AST level, and DRI, while recipient-related covariates included age, sex, BMI, model for end-stage liver disease score, functional status score, race, diabetes, and cause of liver failure. After PSM, the incidence of PNF, and the 30- and 90-day graft and patient survival rates were observed to be comparable between the CA and non-CA groups (each, n = 7157).

## RESULTS

### Donor and Recipient Characteristics

A total of 115,202 cases were included in the study, of which 7364 and 107,838 were classified as the CA and non-CA groups, respectively. The median follow-up time was 6.5 ± 5.1 years. After 1:1 PSM, 7157 patients were included in each of the groups. The general characteristics of the groups pre- and post-PSM are shown in Supplemental Table 2, http://links.lww.com/AOSO/A434. CA donors were observed to be significantly younger compared to non-CA donors (40 ± 16 years vs. 42 ± 16 years, *P* < 0.001). While CA donors demonstrated significantly higher serum creatinine and transaminase levels, the total bilirubin levels were significantly lower (0.87 ± 0.80 mg/dL vs. 0.93 ± 1.40 mg/dL, *P* = 0.001).

The average cold ischemia time was significantly shorter in the CA group (6.5 ± 2.8 hours vs. 6.7 ± 2.9 hours, *P* = 0.001). CA donors further demonstrated a significantly higher prevalence of diabetes and hypertension but with significantly lower DRI (1.40 ± 0.35 vs. 1.44 ± 0.38, *P* < 0.001). Recipients of the CA group demonstrated slightly but significantly shorter postoperative hospital stay (15.5 ± 20.0 days vs. 16.2 ± 21.3 days, *P* = 0.041).

### Impact of CA on Graft and Recipient Survival

The CA group demonstrated better patient and graft survival outcomes, as shown in Table 1. The incidence of EGF was 412 (5.8%) and 441 (6.2%) in the CA and non-CA groups, respectively (*P* = 0.161). In addition, the CA group demonstrated significantly better graft survival rates at 3 months (95% vs. 94%, *P* < 0.001), 1 year (90% vs. 88%, *P* < 0.001), 5 years (76% vs. 74%, *P* < 0.001), and 10 years (61% vs. 58%, *P* < 0.001). Similarly better patient survival rates were observed. No significant differences in the cause of graft and patient death were observed between the groups.

**TABLE 1.** Rate of EGF, Graft Survival, and Patient Survival, and Cause of Graft Failure and Patient Death

	CA (n = 7157)	Non-CA (n = 7157)	*P*
Outcome			
EGF	412 (5.8%)	441 (6.2%)	0.161
3-month graft survival	95%	94%	<0.001
1-year graft survival	90%	88 %	<0.001
5-year graft survival	76%	74%	<0.001
10-year graft survival	61%	58%	<0.001
3-month patient survival	96%	95%	<0.001
1-year patient survival	92%	90%	<0.001
5-year patient survival	78%	75%	<0.001
10-year patient survival	63%	60%	<0.001
Cause of graft failure			
Primary nonfunction	96 (1.3%)	102 (1.4%)	0.721
Recurrent hepatitis	6 (0.1%)	5 (0.1%)	1.000
Rejection	32 (0.5%)	49 (0.7%)	0.074
Infection	44 (0.6%)	47 (0.7%)	0.833
Thrombosis	53 (0.7%)	44 (0.6%)	0.362
Cholangiopathy	28 (0.4%)	31 (0.4%)	0.698
Others	27 (0.4%)	68 (1.0%)	<0.001
Cause of patient death			
Primary graft failure	286 (4.0%)	346 (4.8%)	0.015
Cardiovascular and Cerebrovascular	288 (4.0%)	324 (4.5%)	0.148
Organ failure	286 (4.0%)	351 (4.9%)	0.008
Hemorrhage	63 (0.9%)	63 (0.9%)	1.000
Infection	248 (3.5%)	305 (4.3%)	0.015
Malignancy	344 (4.8%)	332 (4.6%)	0.665
Others	574 (8.0%)	675 (9.4%)	0.003

**FIGURE 2.**
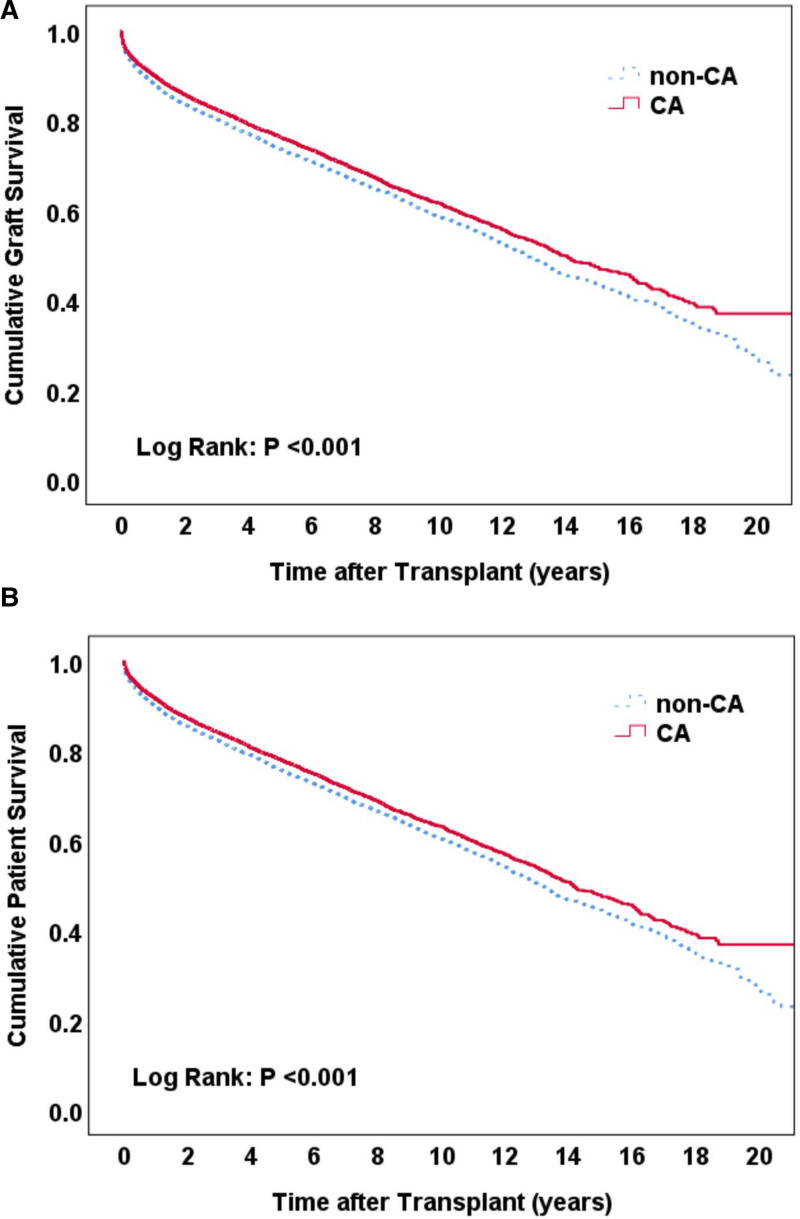
Kaplan–Meier curve analysis on the impact of CA on graft (A) and patient (B) survival.

On Kaplan–Meier survival curve analysis, the CA group demonstrated significantly higher graft and patient survival rates (log-rank *P* < 0.001) (Fig. 2A, B, respectively).

### Independent Predictors of Graft and Recipient Outcomes

Results of the univariate and multivariate regression analyses for factors associated with graft survival are shown in Supplemental Table 3, http://links.lww.com/AOSO/A434. In the univariate analysis, history of CA (HR = 0.93; 95% CI = 0.88–0.98; *P* = 0.016), recipient age (HR = 1.01; 95% CI = 1.01–1.02; *P* < 0.001), diabetes (HR = 1.25; 95% CI = 1.18–1.34; *P* < 0.001), previous abdominal surgery (HR = 1.06; 95% CI = 1.01–1.13; *P* = 0.037), recipients from home (HR = 0.94; 95% CI = 0.91–0.98; *P* = 0.003), as well as donor age (HR = 1.01; 95% CI = 1.01–1.01; *P* < 0.001), diabetes (HR = 1.18; 95% CI = 1.08–1.29; *P* < 0.001), hypertension (HR = 1.19; 95% CI = 1.12–1.26; *P* < 0.001), cold ischemia time (HR = 1.02; 95% CI = 1.01–1.03; *P* < 0.001), and DRI (HR = 1.46; 95% CI = 1.35–1.58; *P* < 0.001) significantly associated with graft survival.

After adjusting for confounding factors, the history of CA demonstrated no significant impact on graft survival (HR = 0.96; 95% CI = 0.90–1.02; *P* = 0.179). However, recipient age (HR = 1.01; 95% CI = 1.01–1.02; *P* < 0.001), diabetes (HR = 1.20; 95% CI = 1.12–1.29; *P* < 0.001), previous abdominal surgery (HR = 1.07; 95% CI = 1.01–1.15; *P* = 0.029), recipients from home (HR = 0.88; 95% CI = 0.83–0.93; *P* < 0.001), as well as donor age (HR = 1.00; 95% CI = 1.00–1.01; *P* = 0.047), cold ischemia time (HR = 1.01; 95% CI = 1.01–1.02; *P* = 0.004), and DRI (HR = 1.29; 95% CI = 1.13–1.47; *P* < 0.001) remained independent associated factors for graft survival. Similar results were observed for patient survival, as detailed in Supplemental Table 4, http://links.lww.com/AOSO/A434.

### Subgroup Analysis According to CAT

**FIGURE 3.**
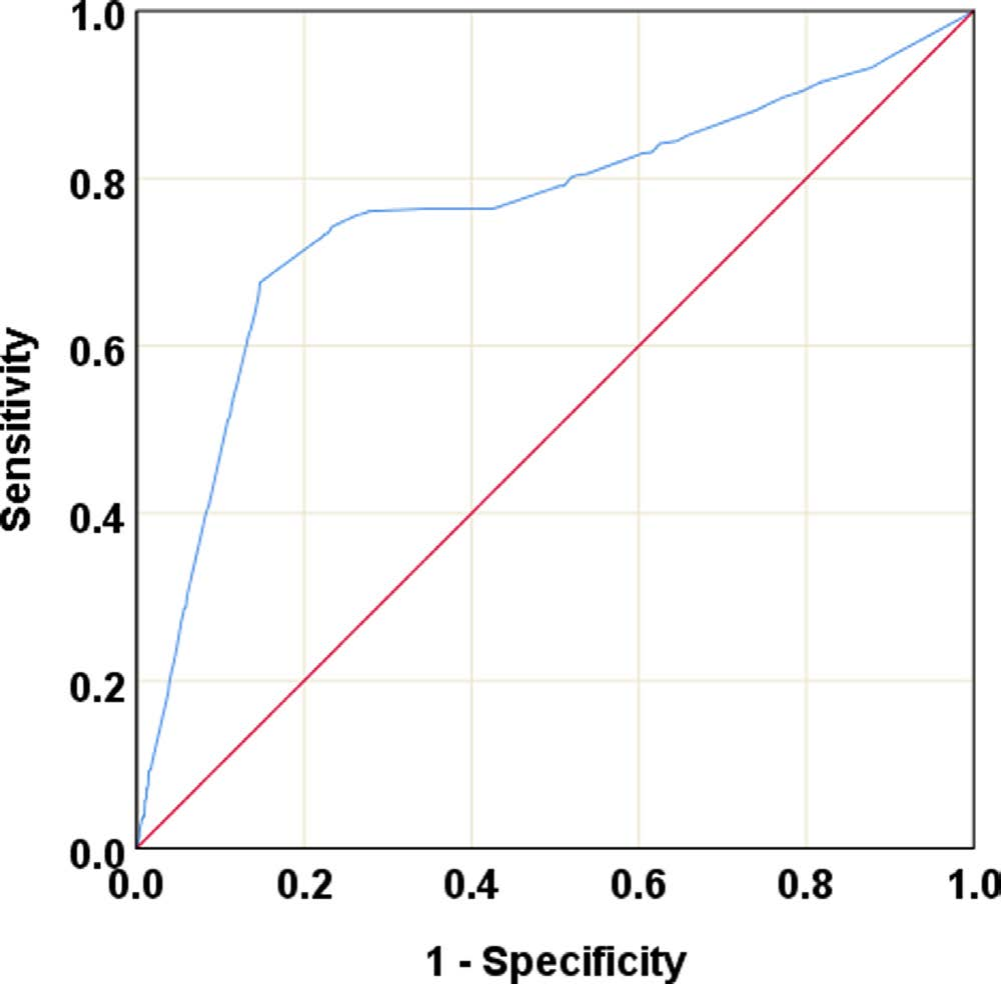
ROC curve analysis on the effects of cardiac arrest time (CAT) on the incidence of early graft failure (EGF).

In the univariate analysis, CAT was noted to correlate with an increased incidence of EGF (OR = 1.03; 95% CI = 1.02–1.04; *P* < 0.001), with an area under the curve of 0.76 (95% CI = 0.74–0.78; *P* < 0.001) on receiver operating characteristic (ROC) curve analysis (Fig. 3). When the cutoff time was set to 30 minutes, the sensitivity and specificity of CAT for predicting EGF were 73% and 86%, respectively, and a maximum Youden index (sensitivity + specificity − 1) was obtained.

Patients were subsequently classified according to this cutoff, as shown in Supplemental Table 5, http://links.lww.com/AOSO/A434. The CAT >30 minutes group demonstrated significantly higher proportion of donors with diabetes (15.6% vs. 10.9%; *P* < 0.001) and hypertension (35.0% vs. 30.2%; *P* < 0.001). In terms of recipients, the CAT >30 minutes group demonstrated significantly higher rates of preoperative portal vein thrombosis (13.8% vs. 11.3%; *P* = 0.004). Postoperatively, recipients of the CAT >30 minutes group demonstrated significantly higher serum creatinine (2.21 ± 2.12 mg/dL vs. 1.74 ± 1.87 mg/dL; *P* < 0.001), blood urea nitrogen (30.4 ± 23.6 mmol/L vs. 24.7 ± 21.8 mmol/L; *P* < 0.001), ALT (153 ± 270 U/L vs. 109 ± 230 U/L; *P* < 0.001), and AST (140 ± 214 U/L vs. 109 ± 236 U/L; *P* < 0.001) levels compared with the CAT <30 minutes group, but with significantly lower total bilirubin levels (0.79 ± 0.64 mg/dL vs. 0.89 ± 0.84 mg/dL; *P* < 0.001). Regarding prognosis, the CAT <30 minutes group demonstrated significantly lower rates of EGF (5.0%), compared with an incidence of 7.8% from the CAT >30 minutes group and 6.2% of the non-CA group (*P* < 0.001).

**FIGURE 4.**
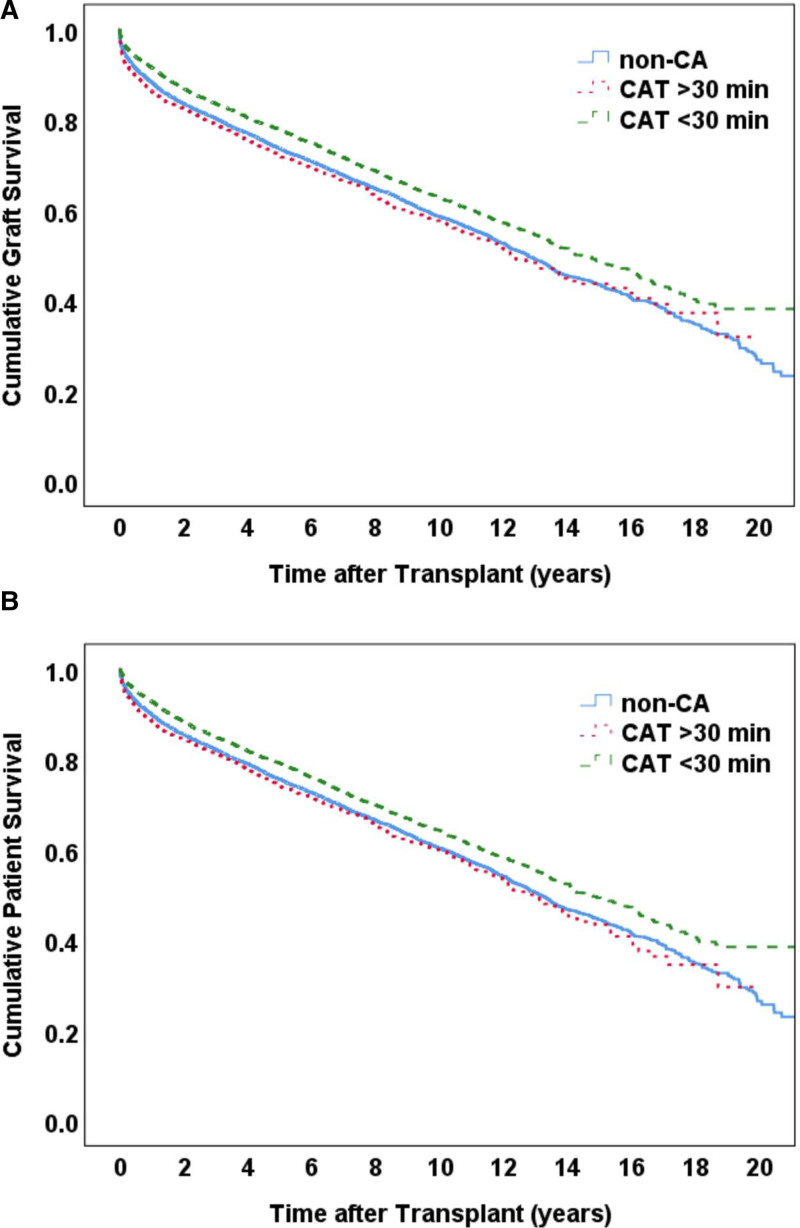
Kaplan–Meier curve analysis on the impact of CAT on graft (A) and patient (B) survival.

The CAT <30 minutes group was found to demonstrate significantly higher graft survival rates compared with the CAT >30 minutes and non-CA groups, with no significant difference observed between the latter 2 groups (Fig. 4A). Similar results were achieved with patient survival rates (Fig. 4B).

## DISCUSSION

This retrospective study of the large-sample OPTN/SRTR database observed that liver transplantation from donors with a history of resuscitated CAs is safe and feasible, with outcomes comparable to conventional DBD liver transplantation within the 10-year follow-up, suggesting its potential as a safe and effective organ source for LT. Our subgroup analysis further identified 30 minutes as the cutoff duration for CAs to confer the clinical benefits of ischemic preconditioning.

Our study results support the use of livers from DBDs who have experienced brief CA events, which is of great significance for expanding the marginal donor organ pool and addressing the current issues of organ shortage. The traditional view holds that prolonged warm ischemia as a result of CA, coupled with hemodynamic instability and metabolic disturbances, increases the risk of reperfusion injuries in transplanted grafts. This study found comparable graft and recipient survival rates between donors with and without a history of CA. Although recipients of CA livers demonstrated significantly higher transaminase levels, suggesting greater degrees of liver injury, they showed a significantly lower incidence of early graft dysfunction. Our findings are in corroboration with those of Levesque et al,^18^ who conducted a retrospective analysis involving 165 LT recipients, of whom 34 (20.6%) were from DBDs with CA before organ procurement (median CAT, 15 minutes). They found that a history of CA had no impact on the incidence of graft failure, PNF, and postoperative complications, or on graft and patient survival rates at 6-month follow-up. Similarly, the retrospective analysis by Totsuka et al,^9^ wherein 37 (20.4%) of the 181 patients underwent LT from DBDs with reversible CA, found significantly lower peak ALT levels post-transplantation among recipients of CA donors (718 IU/L vs. 1507 IU/L, *P* < 0.05), despite significantly higher transaminase levels before organ procurement. Altogether, these findings support the notion that reversible CA in donors may produce certain protective effects on newly transplanted livers through adaptive ischemic preconditioning responses. However, some studies reached the opposite conclusion that reversible CA in donors had no protective effect on grafts.^22^

Downtime duration was found as the key influencing factor of patient prognosis. Our findings highlighted that donors with CAs of <30 minutes achieved significantly better graft and patient outcomes compared to not only those with prolonged CAs but also those without a history of such complications. ROC curve analysis further demonstrated that a downtime cutoff of 30 minutes possesses high sensitivity and specificity for predicting EGF. This suggests the clinical importance of effective control of CAs to ensure the quality of donor livers. Prolonged CA may exacerbate ischemic damage to the donor liver and compromise its ability to tolerate reperfusion, ultimately leading to an increased risk of graft dysfunction and failure. Based on our results, it can thereby be speculated that CAs limited to 30 minutes may trigger similar endogenous protective processes, and should be deemed an acceptable donor criteria for LT. Levesque’s study also has shown that ischemic preconditioning can activate a series of protective pathways to ameliorate the degree of ischemic-reperfusion injuries and prevent pathological processes such as mitochondrial dysfunction, apoptosis, inflammatory response, and microcirculatory disorders.^18^ Attempts to emulate the protective effects of this adaptive pathophysiological process have resulted in the emergence of different conditioning strategies, such as direct ischemic preconditioning, ischemic postconditioning, and remote ischemic preconditioning, which have shown promising results.^23–27^

Donor quality is considered a key factor affecting the prognosis of LT. Currently, the DRI is the commonly used standardized assessment tool for the quality of donor livers in clinical practice. This index integrates multiple risk factors such as donor age, race, height, cause of death, allocation type, and cold ischemia time.^21,28^ However, the DRI does not take into account CA events. This study found that under similar DRI conditions, DBD livers with CAs of <30 minutes demonstrated better graft and patient survival outcomes than DBD livers without any history of CA, suggesting that DRI scores alone may underestimate the quality of donor livers subjected to brief CAs. ROC curve analysis confirmed that downtime was an independent prognostic factor for DBD liver transplantation. Therefore, supplementing DRI with data on CA duration is expected to further improve the donor evaluation system to allow for more accurate screening of high-quality marginal donor livers and minimize organ waste. In considering the use of such donor livers, a comprehensive evaluation of factors such as recipient conditions, perioperative management, and transplant center experience, as well as careful weighing of the risks and benefits, would be warranted.

Considering the poor transplant outcomes observed with CAs of >30 minutes, this may be defined as a marginal donor liver. Such donor livers were observed to generally develop more severe ischemic liver injuries, manifesting as significantly elevated transaminase, creatinine, and urea nitrogen levels. Significantly lower total bilirubin levels were further demonstrated, which may reflect reduced bile stasis as a result of hepatocyte necrosis. In addition, donors with prolonged CAs were older, had higher BMI, and had greater comorbidities, such as diabetes and hypertension, suggesting poorer overall tissue and organ reserve. Therefore, timely identification and management of these high-risk factors and the optimization of donor status are crucial for improving prognosis. Strict evaluation of livers of DBDs with reversible CA, with a comprehensive review of donor age, complications, laboratory results, and gross organ status are thereby of great clinical significance.

This study had several limitations. First, given its retrospective design, the effects of confounders and selection bias could not be completely excluded. While PSM was performed, there may still be a possibility of unknown confounding variables. Second, due to the lack of pathological data such as the degree of steatosis and fibrosis in the SRTR database, it was impossible to characterize the pathological changes of CA DBD livers or evaluate their impact on prognosis. Finally, post-LT immunosuppressive and antiviral regimens were largely heterogeneous across transplant centers and may have affected our results. So large-sample randomized controlled trials to verify our results are needed in our future works.

In summary, this study showed that with strict evaluation and screening, the use of liver grafts from DBDs with a history of CA (CAT <30 minutes) is safe and effective, with outcomes similar to those of liver grafts from DBDs without a history of CA. These findings suggest that donor livers from resuscitated CA can potentially be noninferior to donor livers procured from DBD without a history of CA, provided that strict selection criteria are applied. This opens up a promising future for the use of liver grafts from resuscitated CA donors, potentially expanding the pool of available organs for transplantation.

## Supplementary Material
