# Supplementary material for: Impact of Resuscitated Cardiac Arrest in the Brain-dead Donors on the Outcome of Liver Transplantation: A Retrospective and Propensity Score Matching Analysis
Source: Ann Surg Open. 2024 Nov 25;5(4):e522. doi: 10.1097/AS9.0000000000000522 (PMC11661731; doi:10.1097/AS9.0000000000000522)
Supplement: Supplementary file 1 [file as9-5-e522-s001.pdf]

Supplemental Table 1. Unmatched cohort univariable and final multivariable logistic regression model based on EGF.

	Univariable Model			Multivariable Model		
	OR	95% CI	*p*	OR	95% CI	*p*
CA (vs. non-CA)	0.894	0.810-0.987	0.027	0.869	0.766-0.985	0.025
Donor age	1.007	1.006-1.009	<0.001	1.001	0.999-1.004	0.297
Donor gender (male)	0.849	0.811-0.890	<0.001	0.947	0.892-1.005	0.075
Donor BMI	0.998	0.995-1.002	0.319	1.004	1.000-1.008	0.075
Donor last AST	1.000	1.000-1.000	0.013	1.000	1.000-1.000	0.029
Donor last ALT	1.000	0.999-1.000	<0.001	1.000	0.997-1.000	0.025
Donor last bilirubin	1.022	1.007-1.037	0.003	1.050	1.026-1.074	<0.001
Donor last sodium	0.999	0.996-1.002	0.540	1.000	0.997-1.003	0.935
DRI	1.685	1.591-1.784	<0.001	1.477	1.320-1.653	<0.001
Partial allograft	1.300	1.082-1.563	0.005	1.205	0.943-1.539	0.137
CIT	1.063	1.057-1.070	<0.001	1.056	1.048-1.064	<0.001
Recipient age	1.000	0.997-1.002	0.749	1.005	1.003-1.008	<0.001
Recipient gender (male)	0.885	0.844-0.929	<0.001	0.971	0.915-1.030	0.324
Recipient BMI	1.011	1.007-1.014	<0.001	1.014	1.009-1.018	<0.001
Recipient medical condition (not hospitalized)	0.679	0.660-0.699	<0.001	0.667	0.637-0.700	<0.001
Recipient PVT	1.399	1.305-1.499	<0.001	1.491	1.380-1.609	<0.001
Recipient SBP	1.430	1.306-1.566	<0.001	1.280	1.140-1.436	<0.001
Previous abdominal surgery	1.129	0.982-1.297	0.088	1.038	0.801-1.346	0.777
MELD	1.018	1.016-1.021	<0.001	1.000	0.996-1.004	0.958

EGF: early graft failure; OR: Odds ratio; CI: Confidence interval; CA: Cardiac arrest; BMI: Body mass index; AST: Aspartate aminotransferase; ALT: Alanine aminotransferase; DRI: Donor risk index; CIT: Cold ischemia time; PVT: Portal vein thrombosis; SBP: Spontaneous bacterial peritonitis; MELD:

Model for End-Stage Liver Disease.

Supplemental Table 2. Donor and patient demographic and clinical outcome between recipients of brain-dead donors with and without cardiac arrest

	Unmatched			PSM		
	CA (n = 7,364)	non-CA (n = 107,838)	*P*	CA (n = 7,157)	non-CA (n = 7,157)	*P*
Recipient						
Age: years	55 ± 11	54 ± 11	0.102	55 ± 11	54 ± 11	0.121
Male (%)	4,874 (66.2)	71,337 (66.2)	0.740	4,734 (66.1)	4,757 (66.4)	0.684
BMI: kg/m^2	28.62 ± 6.00	28.56 ± 6.00	0.360	28.63 ± 5.99	28.68 ± 6.13	0.658
Race (%)						
Caucasian	5,371 (72.9)	76,674 (71.1)	0.062	5,213 (72.8)	5,118 (71.5)	0.076
African-American	742 (10.1)	9,597 (8.9)	0.051	725 (10.1)	624 (8.7)	0.064
Hispanic	880 (12.0)	15,324 (14.2)	<0.001	854 (11.9)	972 (13.6)	0.132
Asian	273 (3.7)	4,795 (4.4)	0.073	269 (3.8)	342 (4.8)	0.096
Others	98 (1.3)	1,448 (1.3)	0.994	96 (1.3)	101 (1.4)	0.774
Diagnosis (%)						
HCV cirrhosis	1,636 (22.2)	23,273 (21.6)	0.203	1,564 (21.9)	1,532 (21.4)	0.516
HBV cirrhosis	187 (2.5)	3423 (3.2)	0.052	178 (2.5)	240 (3.4)	0.112
NASH	1,197 (16.3)	16,911 (15.7)	0.191	1,172 (16.4)	1,115 (15.6)	0.352
ALD	1,796 (24.4)	25,813 (23.9)	0.382	1,753 (24.5)	1,772 (24.8)	0.783
HCC	1,076 (14.6)	16,927 (15.7)	0.103	1,070 (15.0)	1,088 (15.2)	0.872
Others	1,472 (20.0)	21,491 (19.9)	0.905	1,420 (19.8)	1,410 (19.7)	0.913
MELD score	22 ± 10	22 ± 10	0.110	22 ± 10	22 ± 10	0.124
Pretransplant condition (%)						
In ICU	878 (11.9)	15,559 (14.4)	<0.001	834 (11.7)	1,066 (14.9)	<0.001
Hospitalized not in ICU	1,354 (18.4)	20,061 (18.6)	0.653	1,326 (18.5)	1,377 (19.2)	0.276
Not Hospitalized	5,132 (69.7)	72,218 (66.9)	<0.001	4,997 (69.8)	4,714 (65.9)	<0.001
Hypertension (%)	1,025 (13.9)	14,153 (13.1)	0.052	993 (13.9)	994 (13.9)	1.000

Diabetes (%)	1,888 (25.6)	27,428 (25.4)	0.699	1,841 (25.7)	1,835 (25.6)	0.909
Previous abdominal surgery (%)	3,133 (42.5)	44,699 (41.5)	0.067	3,070 (42.9)	2,973 (41.5)	0.101
PVT (%)	872 (11.8)	10,512 (9.7)	<0.001	857 (12.0)	678 (9.5)	<0.001
SBP (%)	389 (5.3)	5,654 (5.2)	0.874	364 (5.1)	376 (5.3)	0.678
Hospital stay: days	15.5 ± 20.0	16.3 ± 21.8	0.007	15.5 ± 20.0	16.2 ± 21.3	0.041
Donor						
Age: years	40 ± 16	42 ± 17	<0.001	40 ± 16	42 ± 16	<0.001
BMI: kg/m^2	28.04 ± 6.76	27.40 ± 6.38	<0.001	28.10 ± 6.77	27.52 ± 6.44	<0.001
Male (%)	4,233 (57.5)	64,442 (59.8)	<0.053	4,126 (57.6)	4,285 (59.9)	0.077
Race (%)						
Caucasian	4,853 (65.9)	70,742 (65.6)	0.603	4,694 (65.6)	4,688 (65.5)	0.930
African-American	1,437 (19.5)	18,942 (17.6)	<0.001	1,415 (19.8)	1,265 (17.7)	<0.001
Hispanic	874 (11.9)	14,409 (13.4)	<0.001	853 (11.9)	971 (13.6)	0.003
Asian	133 (1.8)	2661 (2.5)	<0.001	130 (1.8)	164 (2.3)	0.054
Others	67 (0.9)	1084 (1.0)	0.465	65 (0.9)	69 (1.0)	0.875
Cause of death (%)						
Anoxia	3,769 (51.2)	27,191 (25.2)	<0.001	3,711 (51.9)	1,809 (25.3)	<0.001
CVA/stroke	1,659 (22.5)	41,052 (38.1)	<0.001	1,574 (22.0)	2,751 (38.4)	<0.001
Head trauma	1,808 (24.6)	36,879 (34.2)	<0.001	1,751 (24.5)	2,420 (33.8)	<0.001
Others	128 (1.7)	2,716 (2.5)	<0.001	121 (1.7)	177 (2.5)	0.051
Last creatinine: mg/dl	1.85 ± 1.94	1.60 ± 1.72	<0.001	1.86 ± 1.95	1.61 ± 1.70	<0.001
Last BUN: mmol/L	26.0 ± 22.3	22.2 ± 18.8	<0.001	26.2 ± 22.4	22.5 ± 19.0	<0.001
Last sodium: mmol/L	147 ± 8	148 ± 8	<0.001	147 ± 8	148 ± 8	0.232
Last INR	1.37 ± 1.41	1.36 ± 1.34	0.572	1.37 ± 1.41	1.36 ± 1.16	0.611
Last bilirubin: mg/dl	0.88 ± 1.03	0.93 ± 1.20	<0.001	0.87 ± 0.80	0.93 ± 1.40	0.001
Last ALT: U/L	120 ± 240	74 ± 216	<0.001	121 ± 242	80 ± 548	<0.001
Last AST: U/L	117 ± 229	80 ± 155	<0.001	117 ± 231	80 ± 145	<0.001
CIT: hours	6.5 ± 2.9	6.7 ± 3.0	<0.001	6.5 ± 2.8	6.7 ± 2.9	0.001

Diabetes (%)	883 (12.0)	11,786 (10.9)	0.005	869 (12.1)	772 (10.8)	0.011
Hypertension (%)	2,299 (31.2)	36,990 (34.3)	<0.001	2,252 (31.5)	2,485 (34.7)	<0.001
DRI	1.40 ± 0.35	1.44 ± 0.38	<0.001	1.40 ± 0.35	1.44 ± 0.38	<0.001
Partial allograft (%)	79 (1.1)	1,412 (1.3)	0.091	75 (1.0)	88 (1.2)	0.307

CA: Cardiac arrest; PSM: Propensity score matching; SD: Standard Deviation; BMI: Body mass index; HCV: Hepatitis C virus; HBV: Hepatitis B virus; NASH: non-alcoholic steatohepatitis; ALD: Alcoholic liver disease; HCC: Hepatocellular carcinoma; MELD: Model for end stage liver disease; ICU: Intensive care unit; PVT: Portal vein thrombosis; SBP: Spontaneous bacterial peritonitis; CVA: Cerebrovascular diseases; BUN: Blood urea nitrogen; INR: International normalized ratio; ALT: Alanine aminotransferase; AST: Aspartate aminotransferase; CIT: Cold ischemia time; DRI: Donor risk index.

Supplemental Table 3. Univariate and Multivariate Cox proportional-hazards regression models based on graft survival

Covariates	Univariate analyses		Multivariate analyses	
	HR (95% CI)	P	HR (95% CI)	P
CA (vs. non-CA)	0.93 (0.88-0.98)	0.016	0.96 (0.90-1.02)	0.179
Recipient age	1.01 (1.01-1.02)	<0.001	1.01 (1.01-1.02)	<0.001
Recipient gender (male)	1.03 (0.97-1.09)	0.401	1.04 (0.97-1.11)	0.324
Recipient BMI	0.99 (0.99-1.01)	0.233	0.99 (0.98-0.99)	0.026
MELD	1.00 (0.99-1.01)	0.465	0.99 (0.99-1.00)	0.075
Recipient diabetes	1.25 (1.18-1.34)	<0.001	1.20 (1.12-1.29)	<0.001
PVT	1.07 (0.97-1.18)	0.160	1.04 (0.94-1.15)	0.458
Previous abdominal surgery	1.06 (1.01-1.13)	0.037	1.07 (1.01-1.15)	0.029
Medical condition (not hospitalized)	0.94 (0.91-0.98)	0.003	0.88 (0.83-0.93)	<0.001
Donor age	1.01 (1.01-1.01)	<0.001	1.00 (1.00-1.01)	0.047
Donor gender (male)	0.99 (0.94-1.05)	0.848	1.02 (0.96-1.09)	0.483
Donor diabetes	1.18 (1.08-1.29)	<0.001	1.04 (0.94-1.15)	0.414
Donor hypertension	1.19 (1.12-1.26)	<0.001	1.01 (0.93-1.09)	0.884
CIT	1.02 (1.01-1.03)	<0.001	1.01 (1.01-1.02)	0.004
Partial allograft	0.91 (0.68-1.20)	0.489	0.96 (0.70-1.31)	0.794
DRI	1.46 (1.35-1.58)	<0.001	1.29 (1.13-1.47)	<0.001

HR: hazard ratio; CI: confidence interval; CA: Cardiac arrest; MELD: Model for end stage liver disease; PVT: Portal vein thrombosis; CIT: Cold ischemia time; BMI: body mass index; BUN: blood urea nitrogen; DRI: donor risk index.

Supplemental Table 4. Univariate and Multivariate Cox proportional-hazards regression models based on patient survival

Covariates	Univariate analyses		Multivariate analyses	
	HR (95% CI)	P	HR (95% CI)	P
CA (vs. non-CA)	0.94 (0.89-0.99)	0.044	0.97 (0.91-1.03)	0.327
Recipient age	1.01 (1.01-1.02)	<0.001	1.02 (1.01-1.02)	<0.001
Recipient gender (male)	1.04 (0.98-1.11)	0.229	1.05 (0.98-1.12)	0.206
Recipient BMI	1.00 (0.99-1.01)	0.301	0.99 (0.99-1.00)	0.028
MELD	1.00 (0.99-1.00)	0.973	1.00 (1.00-1.00)	0.358
Recipient diabetes	1.31 (1.23-1.40)	<0.001	1.24 (1.15-1.33)	<0.001
PVT	1.08 (0.98-1.20)	0.124	1.03 (0.93-1.15)	0.569
Previous abdominal surgery	1.08 (1.02-1.15)	0.009	1.09 (1.02-1.16)	0.013
Medical condition (not hospitalized)	0.93 (0.90-0.97)	<0.001	0.87 (0.83-0.93)	<0.001
Donor age	1.01 (1.01-1.02)	<0.001	1.00 (0.99-1.01)	0.103
Donor gender (male)	1.02 (0.96-1.08)	0.609	1.04 (0.97-1.12)	0.224
Donor diabetes	1.16 (1.06-1.27)	0.002	1.03 (0.93-1.15)	0.552
Donor hypertension	1.16 (1.09-1.24)	<0.001	0.99 (0.92-1.08)	0.840
CIT	1.02 (1.01-1.03)	<0.001	1.01 (1.01-1.02)	0.020
Partial allograft	0.87 (0.65-1.17)	0.365	0.91 (0.65-1.26)	0.562
DRI	1.41 (1.30-1.52)	<0.001	1.29 (1.12-1.48)	<0.001

HR: hazard ratio; CI: confidence interval; CA: Cardiac arrest; BMI: body mass index; MELD: Model for End-Stage Liver Disease; PVT: Portal vein thrombosis; CIT: Cold ischemia time; DRI: donor risk index.

Supplemental Table 5. Recipient and donor characteristics between CA Donors with CAT >30 min and CAT <30 min

	CAT <30 min (n = 5,272)	CAT >30 min (n = 1,885)	*P*
Recipient			
Age: years	55 ± 11	55 ± 11	0.956
Male (%)	3,495 (66.3)	1,239 (65.7)	0.671
BMI: kg/m^2	28.58 ± 6.05	28.78 ± 5.85	0.208
Race (%)			
Caucasian	3,849 (73.0)	1,364 (72.4)	0.587
African-American	523 (9.9)	202 (10.7)	0.328
Hispanic	636 (12.1)	218 (11.6)	0.591
Asian	200 (3.8)	69 (3.7)	0.833
Others	64 (1.2)	32 (1.7)	0.129
Diagnosis (%)			
HCV	1,156 (21.9)	408 (21.6)	0.820
HBV	134 (2.5)	44 (2.3)	0.667
NASH	851 (16.1)	321 (17.0)	0.384
ALD	1,311 (24.9)	442 (23.4)	0.224
HCC	757 (14.4)	313 (16.6)	0.020
Others	1,063 (20.2)	357 (18.9)	0.267
MELD score	22 ± 10	21 ± 10	0.075
Pretransplant medical condition (%)			
In ICU	615 (11.7)	219 (11.6)	1.000
Hospitalized not in ICU	1,002 (19.0)	324 (17.2)	0.084
Not Hospitalized	3,655 (69.3)	1,342 (71.2)	0.160
Hypertension (%)	716 (13.6)	277 (14.7)	0.229
Diabetes (%)	1,326 (25.2)	515 (27.3)	0.066
Previous abdominal surgery (%)	2,278 (43.2)	792 (42.0)	0.371

PVT (%)	596 (11.3)	261 (13.8)	0.004
SBP (%)	256 (4.9)	108 (5.7)	0.143
Donor			
Age: years	40 ± 16	41 ± 16	0.010
BMI: kg/m^2	27.81 ± 6.63	28.94 ± 7.09	<0.001
Male (%)	3,085 (58.5)	1,041 (55.2)	0.013
Race (%)			
Caucasian	3,431 (65.1)	1,263 (67.0)	0.134
African-American	1,045 (19.8)	370 (19.6)	0.866
Hispanic	650 (12.3)	203 (10.8)	0.075
Asian	96 (1.8)	34 (1.8)	1.000
Others	50 (0.9)	15 (0.8)	0.671
Cause of death (%)			
Anoxia	2,327 (44.1)	1,384 (73.4)	<0.001
CVA/stroke	1,296 (24.6)	278 (14.7)	<0.001
Head trauma	1,546 (29.3)	205 (10.9)	<0.001
Others	103 (2.0)	18 (1.0)	0.003
Last creatinine: mg/dl	1.74 ± 1.87	2.21 ± 2.12	<0.001
Last BUN: mmol/L	24.7 ± 21.8	30.4 ± 23.6	<0.001
Last sodium: mmol/L	147 ± 8	147 ± 8	0.215
Last INR	1.38 ± 1.58	1.35 ± 0.77	0.509
Last bilirubin: mg/dl	0.89 ± 0.84	0.79 ± 0.64	<0.001
Last ALT: U/L	109 ± 230	153 ± 270	<0.001
Last AST: U/L	109 ± 236	140 ± 214	<0.001
CIT: hours	6.5 ± 2.9	6.5 ± 2.6	0.725
Diabetes (%)	575 (10.9)	294 (15.6)	<0.001
Hypertension (%)	1,593 (30.2)	659 (35.0)	<0.001
DRI	1.40 ± 0.35	1.41 ± 0.33	0.189
Partial allograft (%)	61 (1.2)	14 (0.7)	0.147

Outcomes			
Graft failure: n (%)	1,554 (29.5)	641 (34.0)	<0.001
EGF: n (%)	265 (5.0)	147 (7.8)	<0.001
Hospital stay: days	15.5 ± 20.3	15.6 ± 19.3	0.810

CAT: cardiac arrest time; CA: Cardiac arrest; DBD: Donation after brain death; SD: Standard Deviation; BMI: Body mass index; HCV: Hepatitis C virus; HBV: Hepatitis B virus; NASH: non-alcoholic steatohepatitis; ALD: Alcoholic liver disease; HCC: Hepatocellular carcinoma; MELD: Model for end stage liver disease; ICU: Intensive care unit; PVT: Portal vein thrombosis; SBP: Spontaneous bacterial peritonitis; CVA: Cerebrovascular diseases; BUN: Blood urea nitrogen; INR: International normalized ratio; ALT: Alanine aminotransferase; AST: Aspartate aminotransferase; CIT: Cold ischemia time; DRI: Donor risk index.
